# Findings From the Understanding Patterns of Healthy Aging Among Men Who Have Sex With Men Project

**DOI:** 10.1093/geroni/igaa057.3005

**Published:** 2020-12-16

**Authors:** Mark Brennan-Ing, Michael Plankey, Deborah Gustafson

## Abstract

In 1984, the Multicenter AIDS Cohort Study (MACS) was started to identify factors in the HIV epidemic related to disease risk and treatment progression among gay, bisexual, and other men who have sex with men (MSM) in four urban areas in the US: Baltimore, MD/Washington, D.C.; Chicago, IL; Pittsburgh, PA, and Los Angeles, CA. MACS participants complete biannual study visits involving HIV testing, biometric screenings, and psychosocial data collection. In 2015 a MACS sub-study, the Understanding Patterns of Healthy Aging among MSM Project (HAMSM), was started to better understand resiliencies promoting well-being among MSM age 40 and older, including those with HIV. HAMSM has helped us to understand aging trajectories among MSM, and provides a unique combination of physiological and psychosocial data that can inform efforts to support MSM in healthy aging. This symposium will present emerging findings from the HAMSM study. Our first paper examines the relationships between psychological connection to the gay community (PSOC) and developmental regulatory strategies associated with health behaviors and more positive self-appraisals. The second paper examines how PSOC is related to HIV risk reduction behaviors, and if there are differences in such behaviors based on HIV status. Our third paper considers how self-perceptions of aging (age discrepancy, aging satisfaction) are related to frailty and frailty transitions, and if these relationships differ by HIV status. The final paper examines the relationship of social support to frailty among MSM by HIV status. Implications of these findings for research, policy, and programs targeting MSM will be discussed.

